# Malaria intensity in Colombia by regions and populations

**DOI:** 10.1371/journal.pone.0203673

**Published:** 2018-09-12

**Authors:** Alejandro Feged-Rivadeneira, Andrés Ángel, Felipe González-Casabianca, Camilo Rivera

**Affiliations:** 1 Department of Anthropology, Stanford University, Stanford, CA, United States of America; 2 Department of Urban Management and Design, Universidad del Rosario, Bogotá, Colombia; 3 Department of Mathematics, Universidad de los Andes, Bogotá, Colombia; 4 Department of Mathematics and Statistics, Universidad del Norte, Barranquilla, Colombia; 5 Walmartlabs, Sunnyvale, CA, United States of America; Instituto Rene Rachou, BRAZIL

## Abstract

Determining the distribution of disease prevalence among heterogeneous populations at the national scale is fundamental for epidemiology and public health. Here, we use a combination of methods (spatial scan statistic, topological data analysis and epidemic profile) to study measurable differences in malaria intensity by regions and populations of Colombia. This study explores three main questions: What are the regions of Colombia where malaria is epidemic? What are the regions and populations in Colombia where malaria is endemic? What associations exist between epidemic outbreaks between regions in Colombia? *Plasmodium falciparum* is most prevalent in the Pacific Coast, some regions of the Amazon Basin, and some regions of the Magdalena Basin. *Plasmodium vivax* is the most prevalent parasite in Colombia, particularly in the Northern Amazon Basin, the Caribbean, and municipalities of Sucre, Antioquia and Cordoba. We find an acute peak of malarial infection at 25 years of age. Indigenous and Afrocolombian populations experience endemic malaria (with household transmission). We find that *Plasmodium vivax* decreased in the most important hotspots, often with moderate urbanization rate, and was re-introduced to locations with moderate but sustained deforestation. Infection by *Plasmodium falciparum*, on the other hand, steadily increased in incidence in locations where it was introduced in the 2009-2010 generalized epidemic. Our findings suggest that Colombia is entering an unstable transmission state, where rapid decreases in one location of the country are interconnected with rapid increases in other parts of the country.

## 1 Introduction

Malaria in Colombia has been studied from a variety of disciplines that describe disease patterns with dimensions such as the diversity of the vector [1, 2], characteristics of the parasite [3], social phenomena affecting disease transmission [4, 5], and physical phenomena such as climate, weather and land use, [6–8], just to name some of the most important. Mainly, national and local contexts are well understood for a country that presents unusual diversity of environments and social backgrounds (including vast cultural diversity), which, in turn, represent different characteristics of malaria transmission. In contrast with Sub-Saharan Africa, where malaria is commonly a deadly disease affecting primarily children, Colombia is not considered particularly relevant in malarial disease studies given the relatively low mortality when compared with Sub Saharan Africa. However, malaria in Colombia presents certain characteristics that resemble those observed in Southeast Asia. Colombia was one of the first countries where resistance to chloroquine-based treatment was reported. Varied malaria intensity among segregated and diverse populations inhabiting different and unique environments make Colombia one of the few cases where malaria is endemic and where disease patterns are regionally inconsistent, in contrast to several countries that follow a consistent pattern of infection, or whose segregated vulnerable populations do not differ in their epidemic patterns [9, 10]. This does not mean that other countries have a homogeneous experience of malaria intensity across subpopulations or regions.

Malaria is a complex disease, and factors associated to disease severity and resistance have been reported, yet genetic resistance to malaria is better understood than to any other human infectious disease [11]. However, the strong geographical association between resistance to the pathogen and disease severity remains a major challenge to assess the causality of human genetic resistance [11]. We know from evolutionary theory that two critical factors for selection must occur: 1) a population with genetic diversity has to exist for selection to operate; 2) the trait in question must confer a differential in reproductive value for adaptation to evolve. Because African populations exhibit both high genetic diversity and experience severe malaria, genetic resistance to the pathogen appears to have emerged independently in different foci [12]. However, unlike Africa, Colombia has no record of *de novo* emergence of human genetic resistance to malaria. On the contrary, the parasite appears to have developed resistance to treatment. Until recently, it was unknown whether observed pathogen resistance was the result of selection of adaptive mutant strains under drug pressure, or the spread of resistant strains [13]. Genetic evidence suggests that resistant malaria emerged in at least 4 different geographical foci, consistent with the history of reports of resistant pathogens for *Plasmodium falciparum* in the Thailand-Cambodia border and Colombia in the 1950s, then spreading for two decades to South America, Asia and India, and then to Africa in Kenya and Tanzania in the late 1970s [14–16]. Resistant *Plasmodium vivax* was first reported in Papua-New Guinea in 1989, it is currently present in South East Asia, and South America [16].

Studies have found resistant *Plasmodium vivax* at a rate of 11% in representative samples of all blood smears collected in two endemic geographical regions in Colombia: *Llanos orientales* (Eastern Plains) and Urabá [17], while others have found no evidence of resistant *Plasmodium vivax* forms in the Pacific Coast and the Amazon Basin [18]. However, therapeutic failure rates of *Plasmodium falciparum* (for representative samples of all blood smears collected) have been reported as high as 78% for these same regions [18], and 67% in Antioquia [19]. More recent assessments of malaria prevalence in endemic areas also suggest that uncomplicated malaria due to low parasitemia is one of the biggest challenges for malaria control strategies [5, 20, 21], and studies indicate that the observed differences are not attributable to human genetic traits that confer resistance [22].

Few studies have addressed the epidemiology of malarial infection by regions and populations to explore the role of intensity in the emergence of resistant forms of the parasite. However, the role of Colombia in the global epidemiological context of malaria indicates that the country may present unique characteristics for disease transmission. Mainly, the presence, absence, and most importantly, emergence of resistant forms of the parasite in different regions suggests that isolated and distinct epidemic regions exist within the national boundaries, and such characteristics may play a distinctive role in the evolution of the parasite. Here we address the intensity of malaria by regions and human populations in Colombia, and the degree that the epidemic characteristics between regions affect each other.

One key aspect remains poorly understood about malaria dynamics in Colombia: how many different epidemic regions exist, and how do subpopulations in these regions experience malaria. During our research, we interacted with local health officials who conducted malaria prevention programs at both local and national levels. Each public health official had knowledge and expertise about epidemic dynamics in their specific territorial assignment. However, a lack of systematic approaches hamper the ability to formalize such knowledge. Malaria intensity and the social aspects that condition the transmission of the parasite drive public health interventions. However, the regional designations are yet to be formalized based on analysis of malaria dynamics. Decisions about prevention strategies, and how to target the most vulnerable populations are made based primarily on the expertise of local health officials, as indicated by the different authors [6, 23, 24].

Here we generate a systematic classification of the malarious regions and subpopulations of Colombia, to characterize locations and subpopulations with epidemiological aspects of the parasite. Here we address three basic questions surrounding the intensity of malarial infection by ethnicity and region:

1. Is this population experiencing higher intensity of malarial infection than other regions of the country?
2. Is the parasite persisting endemically within this population?
3. Are the epidemic characteristics of this subpopulation affecting other subpopulations?

Specifically, we analyze eight years of malarial case reports, they are examined for both malaria intensity, synchrony and segregation by ethnicity. First, we employ an outbreak detection algorithm [25] widely used [26–28] to identify clusters in space with outbreaks of malaria. Second, we apply methods from Topological Data Analysis (TDA) [29, 30] to visualize synchronous outbreaks: areas that present similar time patterns of malarial epidemics. Finally, regional case reports are explored with descriptive statistics to analyze the intensity of malaria exposure by ethnicity.

## 2 Background

From John Snow’s seminal study of cholera in London, epidemiology has been a spatial discipline [31]. Geographical disease patterns have been widely described for numerous pathogens and regions. We use three methods to analyze malaria in Colombia: disease clustering, disease visualization, and ecological analysis.

### 2.1 Importance of detecting endemicity

The production of good quality maps to understand and visualize risk of disease transmission is recognized as one of the fundamental tools for malaria control strategies, specifically, understanding the relationship between malaria endemicity and the health impact of malaria [32]. Studies suggest that annual entomological inoculation rates (commonly computed as the product of the daily human biting rate, the sporozoite rates from the caught mosquitoes, and the days per year [33]) in Ghana (100-1000), Kenya (10-60) and Gambia (less than 10) are associated to prevention of all cause childhood mortality rates by insecticide treated bed nets, with efficacy of 17%, 33%, and 63%, respectively [32]. These results suggest that public health policies should vary according to malaria endemicity, since bed nets have been the linchpin of malaria prevention strategies since DDT was discontinued as a viable alternative. Yet, evidence suggests that there are several contexts in which bed nets are not efficient [32]. In locations where malaria is intense, the use of bed nets is less efficient to prevent the burden of the disease.

Due to the scarcity of multi-sited studies across different countries, variation of the relationship between endemicity and overall health remains unknown [32]. However, within country variation of malaria has been subject of numerous studies. One study that addresses such relationship is produced by Omumbo et al. [34], using GIS and malaria case reports to map malaria intensity in Kenya. Their results also question the use of treated bed nets in regions where malaria is intense, because in these communities, bednets are the most inefficient [34].

### 2.2 Analyzing malaria from a spatial point of view

Spatial descriptions of variation in malarial infection within countries has been addressed producing maps of risk of contracting the disease using a variety of methods. For example, [35] produced a more accurate visualization of risk of contracting malaria in Mali, by combining regression analysis with *“kriging”* (i.e., an interpolation method similar to smoothing fitted values) to account for local responses to environmental conditions such as weather, population and other topographic and sociological features. Using those methods, they are able to identify regions where the risk is higher than represented in traditional maps [35]. A similar approach, but based on entomological and demographic geo-coded records, is implemented with a GIS analysis to describe local risk of infection based upon proximity to breeding sites and human populations [36]. Beck [37] implemented a variation of these risk maps by integrating remote sensing data to identify locations of high transmission based on human-vector interaction for a region in Mexico, including variation by season.

Estimating the effect of migration on pathogen loads has been a growing interest of epidemiologists in the past years, and multiple methods have been implemented to address such interaction. A data-driven approach to examine the effect of human mobility on epidemics has been the gravity model, used to evaluate measles outbreaks, both by age-classes and by urban and rural settings [38–40]. The main finding of this approach was that population densities were the main driver of outbreak seasonality across different environments [38–40]. Furthermore, the same group has used nighttime light imagery to estimate the effect of changing patterns of population densities on disease outbreaks [41]. Unfortunately, few comparative studies exist to determine which method is more effective under which conditions and for which diseases. However, the method of nighttime light imagery provides good estimates of mobility of populations without access to phone services, often the most vulnerable populations in terms of disease prevalence.

Although the gravity model has been mostly used for directly transmitted diseases, understanding the effect of human mobility on disease epidemics, and more generally how disease disperses over space and time, has been one of the fundamental questions in contemporary spatial epidemiology.

### 2.3 Malaria in the Americas

Contributing 42% of the cases of malaria in the region, Brazil is one of the most important players of the disease in the Americas [8]. Contrary to the spectrum of infection in Colombia, most cases of malaria in Brazil are both Amazonian and caused by *Plasmodium vivax*, with challenges such as asymptomatic and submicroscopic infections, emergence of drug resistance, and social and environmental factors driving risk of disease over a consistently decreasing area of land [8, 42]. Brazil stands both as an example in terms of innovative and effective measures for malaria control, on the one hand, and of policies that promoted malarial infection epidemics on the other [8, 42, 43]. A recent geospatial assesment in the Brazilian Amazon found that malarial infection is more frequently observed in areas with greater forest cover and close to gold mining, and the observed spatial heterogeneity of disease risk suggests that the Acre region should be prioritized by public health interventions, highlighting the relevance of spatial analysis to inform policy [44].

### 2.4 Synchrony in the disease outbreaks

Two approaches have been used to analyze synchrony of disease outbreaks over space and time, controlling for seasonal and environmental variables. Spectral analysis has been used to describe the association between aggravation of asthma symptoms and temperature or atmospheric contamination levels [45, 46], and the association between air pollution and mortality [46]. This technique has been used to study the effect of climatic variation on cholera [47, 48], malarial epidemics [49], and the seasonality of sexually transmitted diseases (STDs) [50].

Some authors suggest that these methods have limitations, because they can only be used for time-series data in which statistical proprieties do not change over time, yet, epidemic data are inherently complex and non-stationary [46]. Furthermore, evidence suggests that epidemic data characteristics do change over time [46, 51, 52].

The limitations of the spectral decomposition methods led to the implementation of the second technique that is most widespread in understanding disease dynamics over space and time, coupled with climatic and environmental conditions from a non-stationary perspective: wavelets, a method used to show how time-series vary as a function of time and space [46, 53].

Wavelet analysis has been used to study geographical hierarchies of measles epidemics, and the observed effect of vaccination policies over time [54]. Associations between dengue epidemics and El Niño Southern Oscillation (ENSO) have also been documented using this method [55]. Kreppel [56] found an association between ENSO, Indian Ocean Dipole (IOD) and plague dynamics in Madagascar, and [57] found similar effect of those two climatic phenomena on infectious gastroenteritis in Japan. [58] documented that Buruli ulcer is affected by short and long rainfall patterns in French Guiana, as well as stochastic events such as ENSO. The relationship between ENSO and cutaneous leishmaniasis has also been documented for Costa Rica [59]. Jose [60] have studied the changing patterns and seasonality of Australian rotavirus epidemics comparing a multiplicity of methods including wavelet analysis, and detected seasonal biannual and quinquennial periods, yet, a three year epidemic period was also found to be dominant. Spectral analysis confirms that serotype harmonics interact in a complex, non-linear fashion, yielding an observable overall pattern beyond the isolated dynamics of each separate serotype, that is more than the sum of the parts, and inherent dynamics remain unchanged but the amplitude of disease infection is modified [60].

### 2.5 Disease cluster identification

Spatial analysis methods have been applied in disease cluster identification. The main approaches used are: K-cluster analysis, detects global clusters based on each case point [61]; the geographical machine [62] and the scan statistic [25], work by aggregating cases in different areas and performing a hypothesis test based on a Bernoulli null model, with the advantageous difference for the scan statistic that it can perform multiple tests simultaneously. We present an implementation of the scan statistic in this case study. Small, isolated outbreaks of malaria in specific communities have been identified as “discrete mini epidemics”, which represent disease severity by using space-time cluster identification [63]. Disease risk by geographical location has also been implemented as simple logistic regressions that include altitude, and physical coordinates of each individual within a case-control study [64]. The scan statistic method has been used by [65] to identify disease clusters over space and time in a South African region. [66] have also implemented the scan statistic method in China to identify clusters and suggest public health resource optimization. Faires [67] used this method to identify clusters of *Clostridium difficile* over time in Ontario, Canada. Duczma [68] implemented the scan statistic to study Chagas’ disease in Brazil, while [69] do the same for end-stage renal disease (ESRD) in northen France. In Virginia, the increasing burden of Lyme disease was documented using spatiotemporal scan statistics [70]. In all cases, studies were able to identify areas with more cases than expected, highlighting in many cases the relevance of regions that did not present a comparatively higher incidence.

Globally, [71] have used maximum likelihood methods (i.e. a similar approach to the scan statistic) to predict areas where malaria is likely to expand as a result of climate change.

A branch that can also be used for cluster identification is Topological Data Analysis (TDA), which applies ideas from the mathematics area of topology to study high dimensional data by obtaining invariants and useful representations of the shape of the data.

In recent years, TDA has been used to find subgroups of individuals with unique genetic and prognostic profiles of breast cancer [30, 72], political alliances in the congress [30], profiles of basketball players [30], pathogen persistence in soil [73], novel patterns in spinal and brain injury [74], subgroups of individuals with different complications from type 2 diabetes [75], among others. We use TDA to find synchronic outbreak clusters among the data.

## 3 Methods

The analysis for this study was generated from case reports based on active and passive detection methods compiled by the Colombian government. Reports of malaria cases are mandatory. Treatment is provided for free to each case, and a positive test is required to disburse the medication. Data was accessed by requesting a user to query the Sispro database of reported cases by the Ministry of Health.

All cases are laboratory confirmed and geocoded to municipality level. We included data for 1,122 municipalities, that range in area from 1 to 65,786 km^2^; total area sampled was 1’144,385 km^2^. For each municipality, we also analyzed sex and ethnic membership of each case, comprising 3,369 different populations. The ethnic denominations used by the Ministry of Health are:

1. Indigenous.
2. Romani.
3. Raizal.
4. Palenquero.
5. Afro, which includes: black, mulatto, african-colombian and afrodescendant.
6. Other, which consists of predominantly mestizos.

Since the combined total cases with ethnic denomination Romani, Raizal or Palenquero are less than the 0.3% of the database, they are excluded from our analysis.

The distribution of malaria cases and segmentation of the data summarized Table 1. See also the section of additional figures.

**Table 1:** Summary and segmentation of registered malaria cases in Colombia between the years 2007 and 2015. Although distribution of sexes among the Colombian population is almost equal, registered malaria cases are predominantly men. *Plasmodium vivax* has a similar ethnic distribution to the Colombian population and as for *Plasmodium falciparum*, the predominant cases are of afro denomination. [76]

Attribute		Total Cases	Percentage (%)
Sex	Male	228075	63.33
	Female	132075	36.67
Ethnicity	Afro	110333	30.63
	Indigenous	38199	10.60
	Other	211618	58.75
Type of Malaria	*Plasmodium falciparum*	115260	32.00
	*Plasmodium vivax*	244890	67.99
Total		360150	

### 3.1 Clustering

The two main objectives are to determine if malaria outbreaks exist in Colombia, and, if so, to determine their location. To address these objectives, we apply scan statistics to perform a hypothesis test in each municipality, examining whether it presents an outbreak. These approaches have been widely used in epidemiological studies [25], [77], and [78].

The approach to test hypothesis is mainly based on the Bernoulli model of the Kulldorff Scan Statistic developed in [25] using the r-package Spatial-Epi [79].

Given the data aggregated by municipality for 2007-2015, each record is assigned to the centroid of the municipality. Because the set of possible outbreaks (all possible aggregations of neighboring municipalities) is almost unlimited in terms of shape and size, a solution is to approximate this set. In this case, a grid *G* of *N* by *N* was overlaid onto Colombia’s jurisdictional boundaries and then the set of possible outbreaks is limited to all the possible sub rectangles within the grid.

Now, under the Bernoulli model we consider a measurement *m* for each rectangle *R* contained in *G*, where *m(R)* corresponds to an integer and in our specific case, the number of individuals inside the given rectangle. This leads us to assume that there is a rectangle *Z* contained in *G* such that each individual inside *Z* has a probability *p* of being infected, while the individuals outside *Z* have a probability *q*. Let *n_R_* be the number of observed malaria cases inside *R*, so by assuming a Bernoulli model and the following hypothesis for our unknown variables *p* and *q*:

$$\begin{array}{ll} H_{0}: & p=q \end{array}$$ (1)

$$\begin{array}{ll} H_{1}: & p>q \end{array}$$ (2)

we have these possible distributions:

- Assuming *H_0_*:
  $$\begin{array}{c} n_{R}∼Bin\left(m\left(R\right),p\right)\;\forall R\subseteq G \end{array}$$ (3)
- Assuming *H_1_*:
  $$\begin{array}{c} n_{R}∼Bin\left(m\left(R\right),p\right)\;\forall R\subseteq Z\;\;\;\text{and}\;\;\;n_{R}∼Bin\left(m\left(R\right),q\right)\;\forall R\subseteq Z^{C} \end{array}$$ (4)

And hence, under *H_1_*, we have that *Z* is a region with potential malaria outbreak.

Lastly, the third and final step is to establish a measure of density for each subrectangle, in this case the likelihood ratio. This measurement of density has desirable properties to compare different sized rectangles [78]. So, [25] derives the formula for likelihood ratio of a generic region. The scan statistic (*ss*) is defined as the highest density measurements for all subrectangles:

$$\begin{array}{c} ss^{*}=\max_{R}\;ss\left(R\right) \end{array}$$ (5)

$$\begin{array}{c} ss\left(R\right)=p^{n_{R}}{\left(1-p\right)}^{m\left(R\right)-n_{R}}q^{n_{G}-n_{R}}{\left(1-q\right)}^{\left(m\left(G\right)-m\left(R\right)\right)-\left(n_{G}-n_{R}\right)} \end{array}$$ (6)

The local measurement *ss(R)* can be interpreted as the likelihood that subrectangle *R* is an outbreak.

To test the hypothesis represented in Eq 1 a Monte Carlo simulation was used to obtain the histogram of the statistic ss* under the null hypothesis. Finally, it assesses the value of ss* with the observed data. If *p* is not greater than *0.05* under the null model, *H_0_* is rejected and we assume an outbreak.

### 3.2 Synchronous epidemic visualization

The main objectives are to determine whether abnormal behaviors are related across municipalities and finding, if a relationship exists, groups of them that have a similar temporal patterns, independent of their geographical layout. To address these matters we turn to Topological Data Analysis (as mentioned in section 2.5).

We apply the method *Mapper*, introduced in [29], that constructs a representation of the data in the form a graph. This graph allows both the analysis and visualization of the data. The vertices of the graph correspond to local clusters and the interactions between these clusters are encoded on the edges of the graph.

*Mapper* detects phenomena that appear both at large and small scale better than other methods, such as principal component analysis (PCA) and cluster algorithms. *Mapper* can be considered a hybrid method that is doing partial clustering, the regions where the clustering is done is guided by the filter. It is a refinement of clustering and scatterplot methods like PCA.

**Fig 1:**
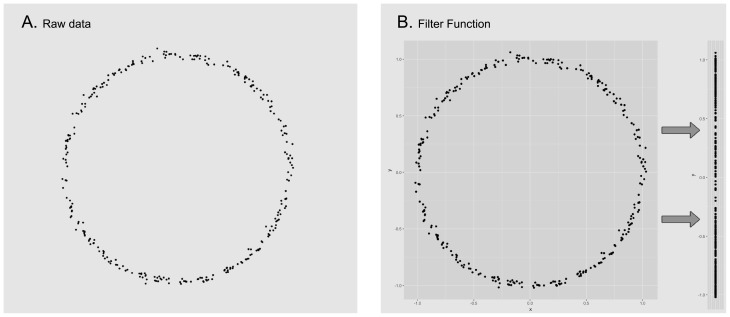
We start with a given data set (image A), for this example the points correspond to a sample of the unitary circle with a small amount of noise. For convenience we will use the euclidean distance to calculate the distance between each pair of points. In the next step, we select the projection onto the *Y* coordinate as our filter function and apply it to the data set (image B).

**Fig 2:**
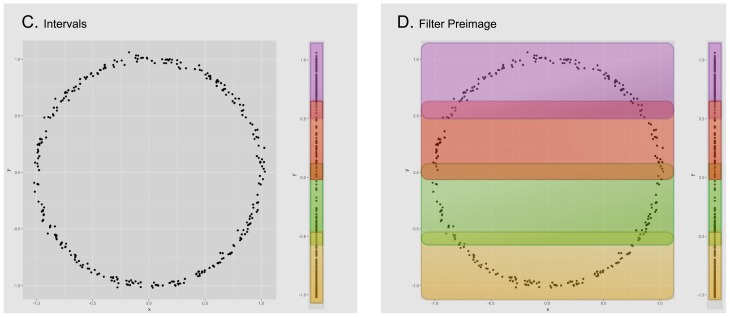
We now divide the image of the data set (under the filter function) into evenly distributed overlapping intervals (image C) and compute the corresponding points in their pre-image (image D). Notice how each pair of overlapping intervals, defines two different subsets of data that can have elements in common.

**Fig 3:**
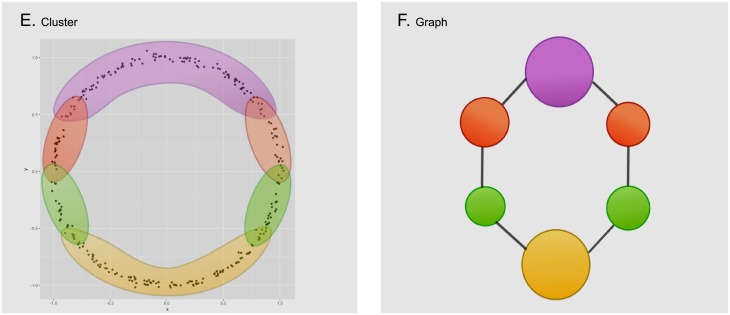
Inside every defined subset of data, we execute a clustering algorithm to detect isolated groups of points (image E). Each of the resulting groups will correspond to a node on the output graph (image F). Notice how nodes are joined together by edges when their corresponding groups have points of the data set in common. Also, the size of the node in the cluster corresponds to the amount of points in its corresponding group.

The input of the method *Mapper* is a collection of points with a notion of similarity and a filter, a function defined on the collection of points. The filter is used to define pieces that cover the collection of points. We apply a clustering algorithm to each piece to obtain a set of local clusters. These are the vertices of the graph. Edges are added to the graph in the following way: two local clusters are connected if they have points in common. Since each vertex and edge correspond to subcollection of points, we can consider the graph to have weights. Figs 1–3 give a visual road map through the *Mapper* algorithm applied to a set sampled from the unitary circle.

In this study, we implement TDA on disease data in Colombia to find topological characteristics that describe spatiotemporal patterns for both *Plasmodium vivax* and *facliparum* separately. We constructed the three *mapper* algorithm’s inputs (collection of points, similarity notion and filter function) as follows, for each incidence type independently:

- **Collection of Points**: For each one of the 1,122 municipalities we calculated an epidemic occurrence vector consisting of binary values for each week from 2007 to 2015. The idea is that each binary value indicates if there was a malaria epidemic on that municipality for the given week. To construct each weekly entry, we executed a Kulldorf Scan Statistic procedure (as explained in the previous section) among all municipalities, but only with data from the given week. So a municipality *k* will have 1 on a certain entry if in the corresponding week it suffered a malaria epidemic (i.e the obtained *p* value from the Kulldorf procedure is not greater than 0.15) or 0 otherwise. We discard municipalities that never showed an outbreak, leaving us with a total of 449 for *Plasmodium vivax* and 269 for *Plasmodium falciparum*.
- **Similarity notion**: Since our collection of points is now a set of binary vectors of dimension 477 (the number of weeks between 2007 and 2015) that represent outbreaks, we computed the similarity between records using the Wasserstein distance. In short, this distance measures the amount of effort required to transform a certain distribution X into another Y. So, if we think of the binary vectors as distributions, this metric captures our intuitive similarity notion among the epidemic records.
  Nevertheless, there is a small shortcoming of using only the Wasserstein metric. When comparing two binary vectors, the metric assumes that they distribute the same amount of mass along all the dimensions, regardless of the amount of positive entries. This means that a binary vector with a single positive entry holds a total *m* mass in that entry, whereas a binary vector with *n* positive entries will hold *m*/*n* mass in each entry. This is a problem because intuitively a binary vector with a single positive entry is very different form a vector with numerous ones, and the mentioned metric does not capture this fact. We solved this problem by adding a factor of the difference of total positive entries among the vectors. Thus, if *X* and *Y* are binary vectors, the similarity notion used in our experiments is:
  $$\begin{array}{c} d\left(X,Y\right)=w\left(X,Y\right)+\frac{\left|\sum X-\sum Y\right|}{\eta } \end{array}$$ (7)
  where *w* is the Wasserstein metric and
  $$\begin{array}{c} \eta =\max_{X,Y}\sum X-\sum Y \end{array}$$
- **Filter Function**: We used a dimension reduction technique called t-SNE [80]. This technique focuses on preserving the probability that points are chosen as neighbors (meaning that they are similar to one another under some notion), while sending them into an euclidean space of lower dimension. For our particular case, we used t-SNE to represent our binary vectors in $R^{3}$, scattered to preserve the Wasserstein metric.

The output of the *Mapper* algorithm is a graph where we select significant subgraphs by disease intensity and number of epidemic weeks. To further visualize the municipalities that appear in the selected subgraphs, we make another graph were the nodes are now the municipalities that appear in nodes of the *Mapper* output and draw connections between those municipalities to visualize them over the Colombian territory.

### 3.3 Ethnicity

Case reports, collected by the national surveillance system and confirmed by laboratory, include components of age and ethnicity in the notification form. This filled form is required by law for every case reported in the country, and treatment to cure the disease is provided by the government for each case. Ethnicity and age are self-reported, and should be interpreted with caution (e.g. no genetic resistance can be inferred, for example). As mentioned before, the three main ethnic groups that were used in this paper are: *Indigenous, Afro* and *Other*.

Data for age and ethnicity were displayed with descriptive statistics to develop patterns of malaria intensity by population for the clusters identified with TDA analysis, and to identify two patterns of intensity. First, the occupational hazard risk profile is characterized by a peak within a particular age-group and contains a pronounced sex difference [42, 81]. Second, an endemic risk profile is characterized by intense malaria exposure at young ages and reduced malaria at later ages, due to overexposure [42, 81, 82].

### 3.4 Social and environmental change

To examine whether epidemic characteristics are associated with land use patterns and development, we visualized deforestation and expansion of the anthropogenic transformation print. To discuss the association between the patterns of malarial infection and deforestation, we plot deforestation alerts published by the government office SIAC [83] for the years available (2013 and 2014). Anthropogenic change, measured using nighttime lights imagery, was visualized using Google Earth Engine to calculate the difference (using map algebra, the difference between two rasters) in stable lights between 1999 and 2013 using an average of 5 years for each pixel for each layer. Mining activities were plotted using the data published by the the United Nations Office on Drugs and Crime [84].

## 4 Results

### 4.1 Clustering

**Fig 4:**
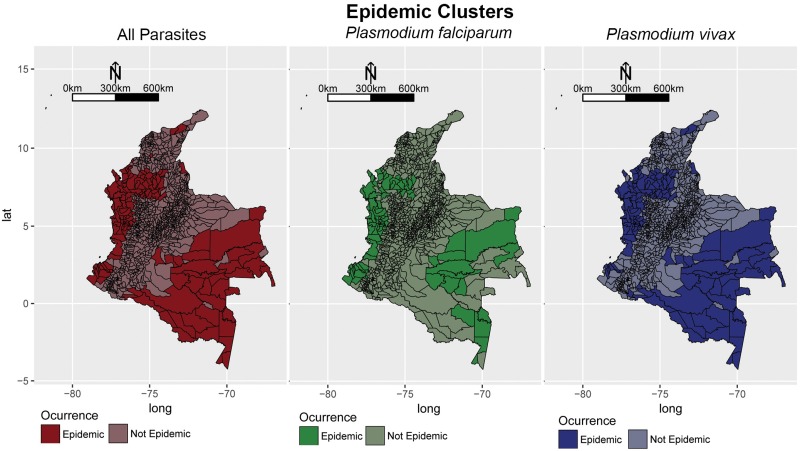
Significant outbreaks of malaria in Colombia from 2007-2015, calculated using the scan statistic developed by [25] based on a likelihood ratio. The significance threshold parameter was calculated using a Bernoulli model where cases were simulated for each municipality, and taking the maximum value. The process was iterated many times and the distribution of the maximum values was calculated to determine the 95% confidence interval.

All significant outbreaks are highlighted in Fig 4. When considering all parasites, the method detects significant outbreaks of malarial infection along the Pacific Coast, the Magdalena river Basin, and the Amazon river Basin. For *Plasmodium falciparum*, significant clusters were observed in municipalities along the Pacific Coast, the border with Panama, and Northen Antioquia (the tertiary Cauca river Basin). The municipalities: Policarpa and Cumbitirá in Nariño, El Retén in Magdalena and Calima in Valle del Cauca appear to be a hidden cluster for this parasite, since they were not marked as epidemic when considering all malarial parasites. And as for *Plasmodium vivax*, significant clusters were observed in municipalities of departments: Cordoba, Vichada and Antioquia. There are also hidden clusters, that appear when only considering this parasite, including: Orito in Putumayo, Piamonte in Cauca and Achi in Bolivar.

### 4.2 Synchronous epidemic visualization

**Fig 5:**
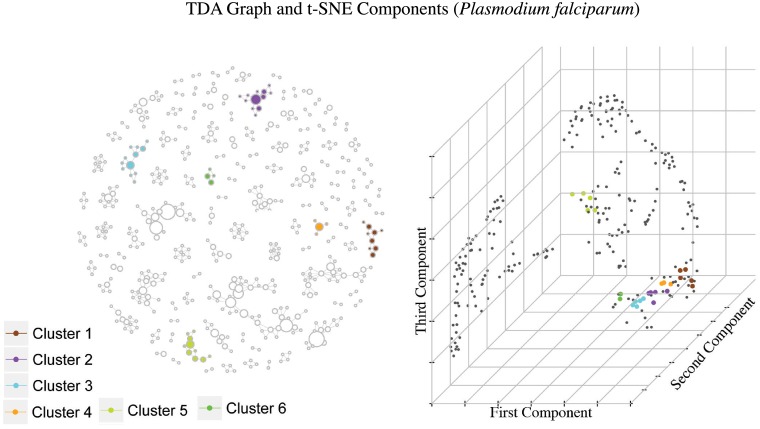
Graph constructed using TDA and t-SNE component plot using the epidemic occurrence vectors, where selected groups have been highlighted. These groups were selected by high overall disease intensity and high epidemic rate. Each cluster can be interpreted as a group of municipalities with *Plasmodium falciparum* incidence that have similar temporal behavior. Notice how the colored dots in the component plot are somewhat grouped together and since these represent municipalities with high epidemic rate, we have highlighted locations with several positive entries in the occurrence vector distributing differently across time.

**Fig 6:**
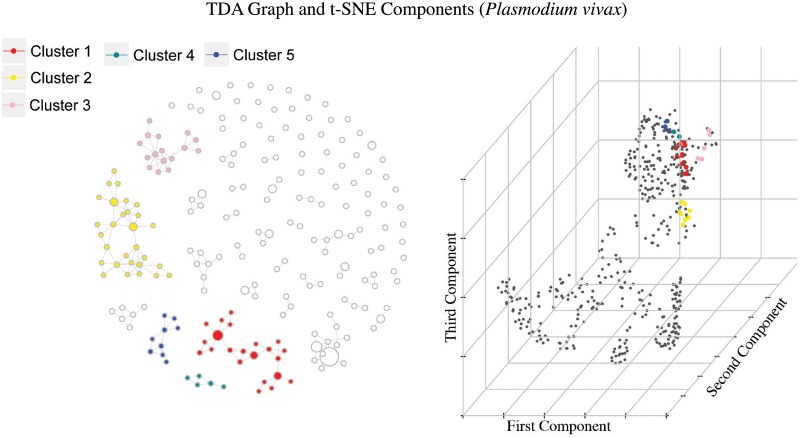
Similar to Fig 5, highlighted groups where selected by high overall disease intensity and high epidemic rate for *Plasmodium vivax*.

TDA enabled us to find at least 11 groups of municipalities with similar temporal behaviors (6 for *Plasmodium falciparum* and 5 for *Plasmodium vivax*) Figs 5 and 6. These groups where selected by high overall disease intensity, defined as the number of cases with infants (age below 5 years) over the total number of cases, or high epidemic rate, understood as the average amount of epidemic weeks among the municipalities in the group.

In the TDA graphs each node represents a group of municipalities. The size of each node will be proportional to the number of municipalities in the group, and two nodes will have an arch between them if they have at least one municipality in common.

**Fig 7:**
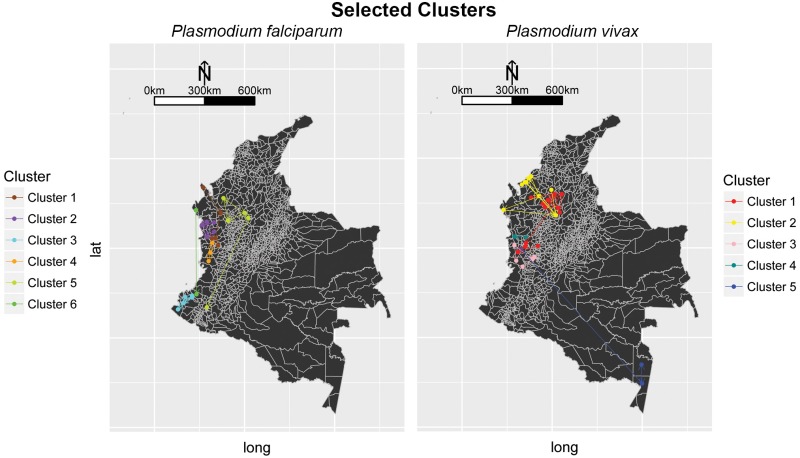
Selected municipalities by TDA over the Colombian territory for both parasites. As expected, the clusters follow some geographic pattern, since the time series where constructed using a Kulldorf procedure that detects clusters geographically. For *Plasmodium falciparum* all clusters are concentrated near the pacific coast and northern Antioquia. Unexpected results happen in cluster 5 for *Plasmodium vivax*, where the grouped municipalities belong to two different geographic regions of the country. This means that the municipalities in this cluster from Chocó and Amazonas have similar time pattern, regardless of their geographical distance.

Now, it is possible to visualize the municipalities appearing in the TDA graph geographically, to get a notion of the part of Colombia the corresponding cluster represents, as seen in Fig 7.

Each of the colored small dots in the maps corresponds to a municipality, contained in some node of the corresponding colored cluster. Notice there are arrows between some points in the map, they represent connections between the municipalities (these connections are not the same as the edges between nodes in the graph). Before we mention how these connections are constructed, let us explain what centrality means for a municipality:

Given a certain municipality, its centrality corresponds to the number of nodes in the graph in which it appears. This means that if we remove a municipality with high centrality, it is very possible that the resulting graph will have less arches and in turn be disconnected.

Now, the connection scheme is as follows: Only municipalities that appear together in several nodes in the TDA graph can have a connection. No municipality will be connected to itself. Municipalities will be connected towards the municipalities in its node with the highest centrality. Note that there could be municipalities with multiple outgoing connections.

We also identified significant municipalities with high centrality in the TDA graphs (Figs 5 and 6) and are reported in Tables 2 and 3. These municipalities are responsible for the connection among several nodes in their corresponding subgraphs appearing in overlapping zones of the selected TDA filter.

**Table 2:** *Plasmodium falciparum*: Selected central municipalities after executing TDA over the epidemic occurrence vectors. These are the municipalities responsible for the connectivity among their respective groups and subgraphs. Quibdó, El Cantón Del San Pablo, Istmina and Roberto Payán appear among the top 10 municipalities with highest epidemic rate and Alto Baudó, Dabeiba and Atrato among the top 10 municipalities with highest disease intensity.

Cluster	Department	Municipality	Rural Pop.	Urban Pop.	Total Pop.
1	Antioquia	Dabeiba	15480	24084	39564
1	Chocó	Atrato	5073	2488	7561
2	Chocó	Quibdó	11752	101134	112886
2	Chocó	Alto Baudó	22739	6222	28961
2	Chocó	Bahia Solano	4864	4230	9094
2	Chocó	Bojayá	5369	4572	9941
3	Nariño	La Tola	2752	5656	8408
3	Nariño	Roberto Payán	16029	863	16892
3	Nariño	San Andres De Tumaco	75366	84668	160034
4	Chocó	El Cantón Del San Pablo	3722	2491	6213
4	Chocó	El Litoral Del San Juan	11186	1058	12244
4	Chocó	Istmina	5319	18320	23639
5	Antioquia	Amalfi	9589	10936	20525
5	Antioquia	Santafe De Antioquia	9267	13636	22903
5	Cordoba	Tierralta	45895	32875	78770
6	Cauca	Guapi	12390	16273	28663

**Table 3:** *Plasmodium vivax*: Selected central municipalities after executing TDA over the epidemic occurrence vectors. These are the municipalities responsible for the connectivity among their respective groups and subgraphs. Cáceres, Nechí and Tadó appear among the top 10 municipalities with highest epidemic rate and Alto Baudo and Medio San Juan among the top 10 municipalities with highest disease intensity.

Cluster	Department	Municipality	Rural Pop.	Urban Pop.	Total Pop.
1	Antioquia	Cáceres	22736	6209	28945
1	Antioquia	Nechí	10228	10440	20668
1	Antioquia	Valdivia	12442	4848	17290
1	Bolívar	Montecristo	9893	7080	16973
1	Chocó	Istmina	5319	18320	23639
1	Chocó	Tadó	6795	11246	18041
2	Antioquia	Vegachi	4824	6469	11293
2	Córdoba	Puerto Escondido	18252	3534	21786
3	Valle del Cauca	Zarzal	12184	28799	40983
4	Chocó	Alto Baudó	22739	6222	28961
5	Chocó	Medio San Juan	8794	4233	13027
5	Amazonas	La Pedrera	3711	0	3711
5	Amazonas	Tarapacá	3775	0	3775

### 4.3 Ethnicity

**Fig 8:**
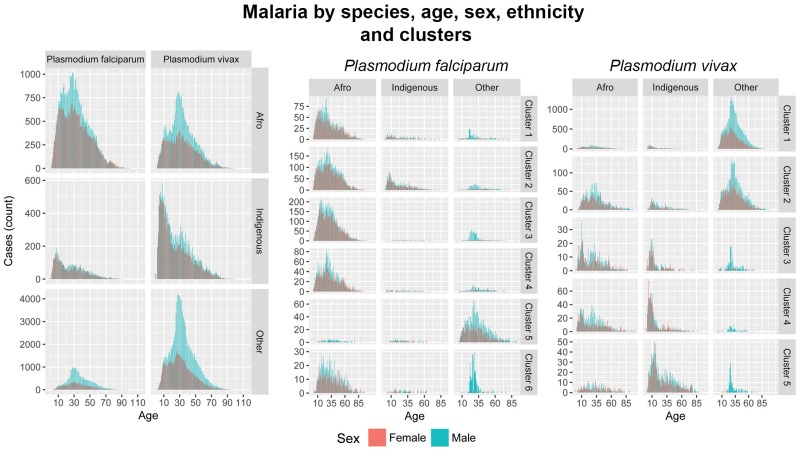
Malaria by parasite species, age, sex, ethnicity and cluster groups of human cases in Colombia. For all parasites, the indigenous ethnic group shows a pattern of endemicity, with most cases being reported for the youngest ages, while people with no ethnic denomination and the Afrocolombian population show a pattern consistent with occupational hazard risk. For *Plasmodium falciparum*, the indigenous and Afrocolombian populations in clusters 1, 2, 3 and 4 suggest that these populations experience intense exposure to malarial infection, with the Afrocolombian population showing occupational hazard transmission. Histograms for the population with no ethnic denomination in all clusters except 4 suggest malarial infection is associated with occupational hazard. And for *Plasmodium vivax*, the indigenous population in clusters 3, 4, and 5 suggest intense exposure to malarial infection among these populations. The population with no ethnic denomination experiences malarial infection as an occupational hazard in all clusters, except 4.

Fig 8 shows the histograms of age reports of malaria by ethnicity and cluster. Two distinctive patterns consistently appearing throughout different regions of Colombia. First, an endemic profile risk (higher density of cases at young ages) was observed for the indigenous populations of clusters 3, 4 and 5 for *Plasmodium vivax*. Second, malarial infection suggesting occupational hazard and intensity of infection among the Afrocolombian populations of clusters 1, 2 and 3 for *Plasmodium falciparum*. Occupational hazard is consistently described for the population with no ethnic denomination across all clusters except cluster 4 for both parasites respectively. In all cases, within the same cluster, we find both endemic and occupational hazard infection patterns across populations segregated by ethnicity. In a histogram of case reports by age and sex, an occupational risk hazard has a unique and characteristic signature: one age class, typically for only one sex, presents an outstanding number of cases compared to any other age class. In the case of malaria in Colombia, we observed that men with no ethnic denomination of ages 20-25 were contracting malaria far more often than any other class. From this simple observation, we inferred the following: first, men of this age class were engaging in activities that posed a risk of contracting the disease. Second, women were not engaging in this activity, nor were men in other age classes. Third, there was no household transmission once they ceased to engage in the risky activity.

**Fig 9:**
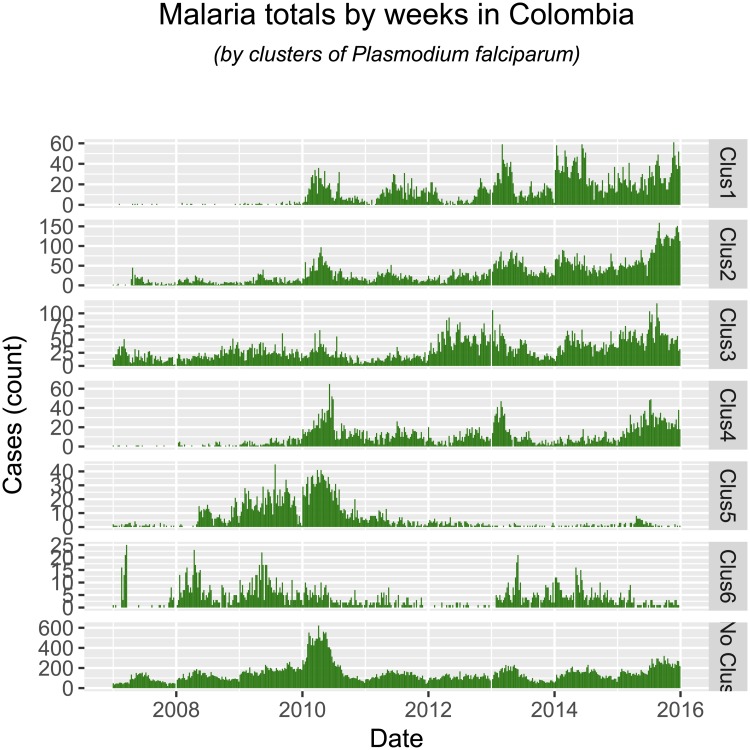
Total cases of *Plasmodium falciparum* by weeks, between the years 2007 and 2015.

**Fig 10:**
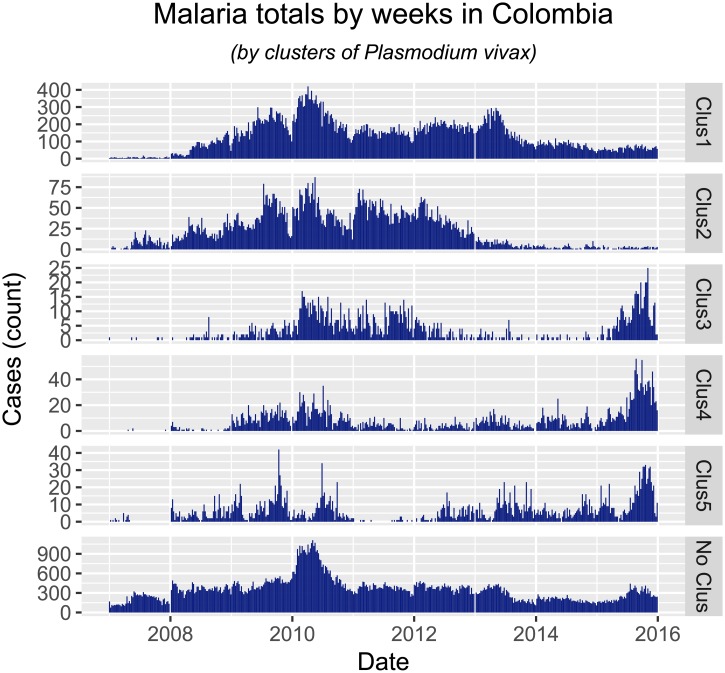
Total cases of *Plasmodium vivax* by weeks, between the years 2007 and 2015.

Most interestingly, our analysis identifies, without explicitly addressing it, locations where the proportion of cases of *Plasmodium falciparum* to *Plasmodium vivax* changed over the past few years Figs 9 and 10. This is one of the open questions in tropical malaria, and our implementation has findings: most of the reduction in *Plasmodium vivax* cases was seen in clusters 1 and 2, while in clusters 3-5 this type of malaria increased recently. Most of the increase in *Plasmodium falciparum* happened in clusters 1-4, in the Pacific Coast (although not explicitly on the coastal areas, but rather in the lower-altitude Andes on the Pacific). Our findings also suggest that the change in ratio of *Plasmodium falciparum* to *Plasmodium vivax* observed at the whole country is comprised of different phenomena, and not just one single change in epidemic characteristics. First, infection by *Plasmodium falciparum* increased in locations where it was not previously present or not persistent (clusters 1-4), while at the same time receding in locations where it was previously a bigger burden (cluster 5, in the lower Cauca Basin). We also find evidence that infection by *Plasmodium vivax* also experienced different epidemic changes across Colombia. First, it receded in locations where it was consistently present (clusters 1-2, lower Cauca Basin and the Caribbean), and increased in locations that did not report cases before the 2009 epidemic (clusters 3-5, Pacific Coast and Amazon Basin). Thus, malarial infection epidemiology in Colombia over the past years has seen the rate of *Plasmodium falciparum* change, we suggest the following stages:

1. 2008-2012: An outbreak of both parasites that peaked in 2010, where *Plasmodium vivax* became the most prevalent parasite, particularly in the lower Cauca Basin and the Caribbean.
2. 2013-2014: A reduction in in the total cases of malarial infection in the whole country, with acute redutions of *Plasmodium vivax* in the Caribbean and the lower Cauca Basin, and an increase of *Plasmodium falciparum* cases in the corridor that connects Acandí and Capurganá with the central Pacific region.
3. 2015-2016: The re-emergence of malarial infection by *Plasmodium vivax* in the central Pacific region and the Amazon.

In other words, the generalized outbreak of malarial infection in 2010 enabled *Plasmodium vivax* to persist in the lower Cauca Basin and the Caribbean, and also introduced the same parasite at lower prevalences in the central Pacific and the Amazon. When the outbreak of *Plasmodium vivax* infection was controlled where it was a bigger burden (lower Cauca Basin and Caribbean), it re-emerged in locations that had not experienced intense malarial infection since the 2010 epidemic (Amazon Basin and central Pacific).

## 5 Further exploration of the results

### 5.1 Deforestation and anthropogenic change

**Fig 11:**
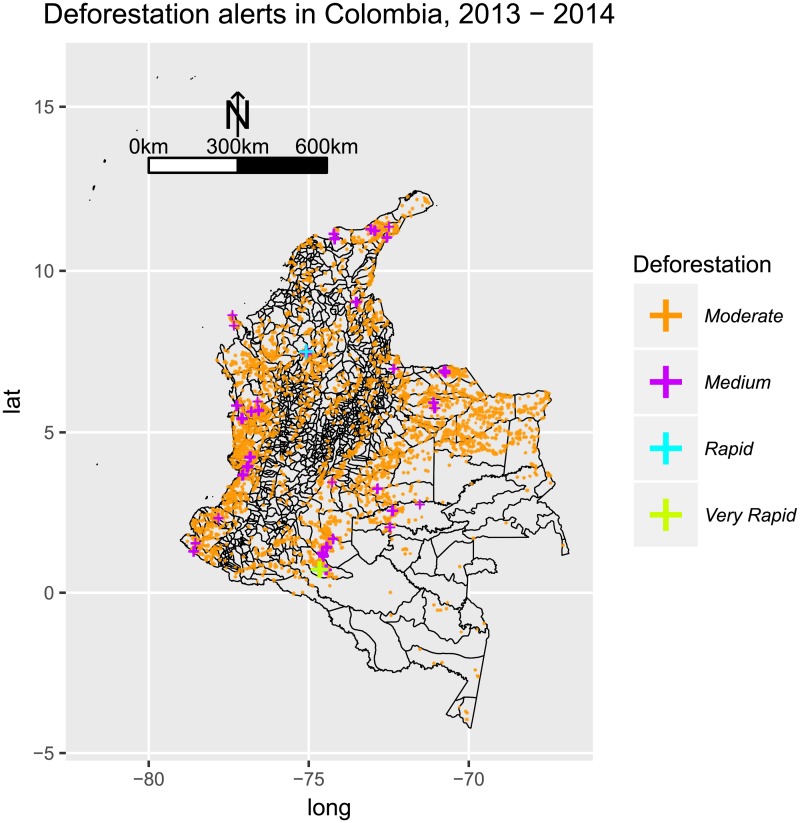
Anthropogenic change in Colombia, 1999-2013, using the nighttime lights dataset NOAA-DMSP-OLS. A mean for 5-year periods was computed for each pixel, and then map algebra was used to calculate the difference between the two periods. Very Rapid anthropogenic change was observed in the region of Bogotá and the Eastern Plains. Rapid change was observed in proximity of the main urban areas along the Andes (Bogotá, Cali, Medellín, and the Coffee Region, the urban areas of the Caribbean, and the Eastern Plains. Moderate and Medium anthropogenic change was observed throughout the Andes and the Caribbean, and the lower Cauca Basin.

All the clusters identified for *Plasmodium falciparum* are located along the Pacific or the lower Cauca Basin, where the human occupation pattern is observed to change little, in comparison to other parts of the country. We find evidence of a different pattern for *Plasmodium vivax*, where clusters 1, 2, and 3 hold both municipalities with low or no change in anthropogenic occupation, but also municipalities with rapid change (Fig 11).

**Fig 12:**
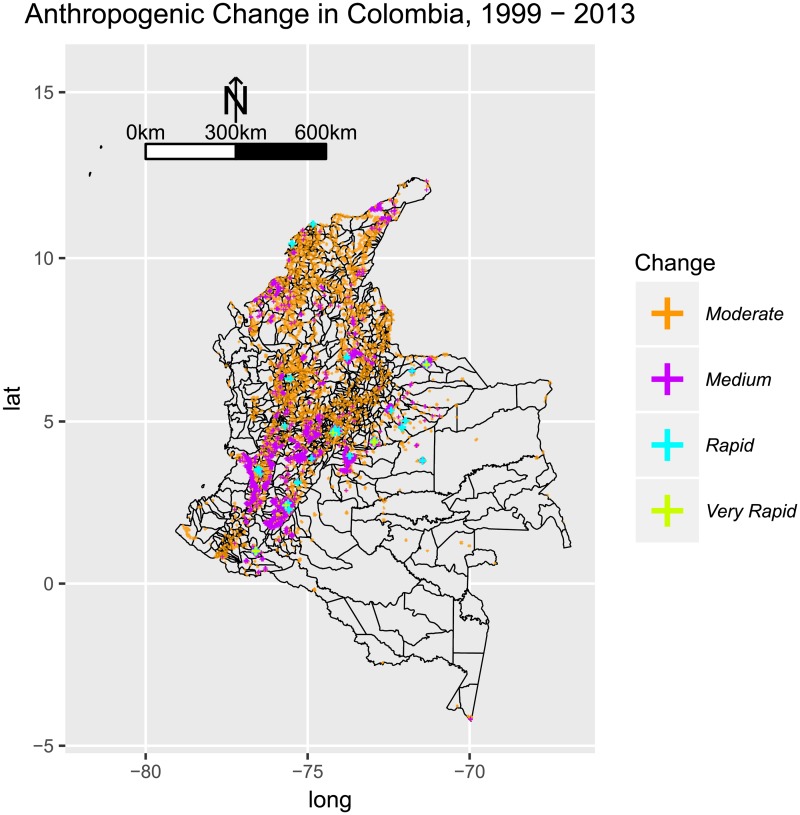
Deforestation alerts in Colombia for years 2013-14, as published by SIAC [83]. Moderate and medium rates of deforestation were observed during the study period along the Pacific Coast, and throughout other parts of the country but more scattered. Rapid deforestation rates were observed in the lower Cauca Basin. Very Rapid deforestation rates were observed in Caquetá, the North Eastern Region of the Amazon Basin.

Deforestation, on the other hand, happened at moderate and medium rates along the Pacific (Fig 12), where clusters of both parasites were observed. Interestingly, the pattern of deforestation along the Pacific is not observed to be the most intense in Colombia, but it is widely spread at lower rates. This suggests that habitat transformation does not need to be intense to harbor malaria transmission, and that a wide-spread moderate deforestation favors the transmission of malaria, perhaps to a greater extent, than rapid deforestation in a single location.

### 5.2 Gold exploitation

**Fig 13:**
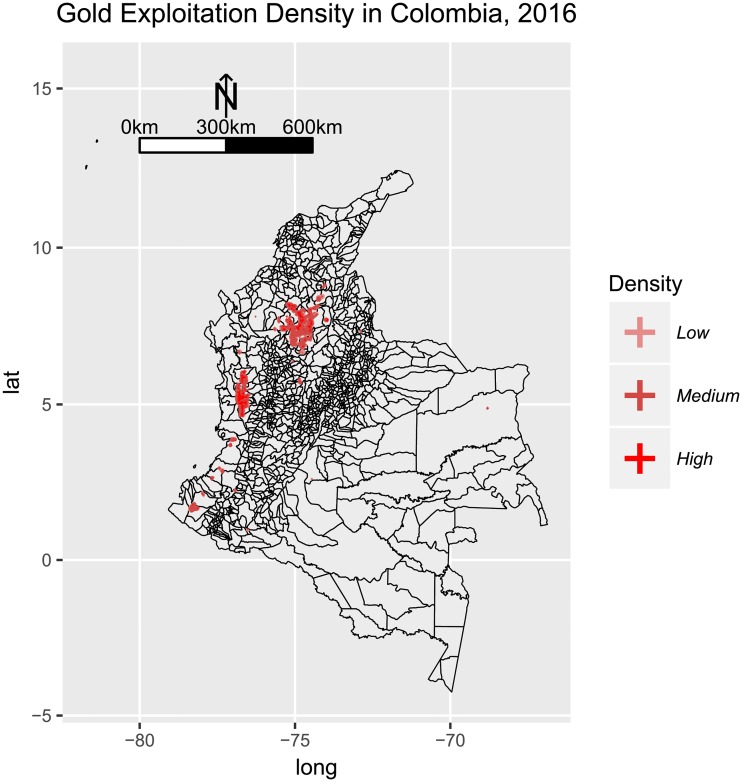
Density for evidence of alluvial gold exploitation in Colombia in 2016, as published by [84]. Three categories are included: Low (less 1 habitant per square kilometer), Medium (between 1.1 and 5 habitants per square kilometer) and High (more than 5 habitants per square kilometer). Evidence of intense mining activities was observed in the lower Cauca and Magdalena Basins, and the the Central Pacific region. Scattered mining activities were observed in the Southern Pacific, some parts of the Eastern Plains.

Our findings suggest a high correlation between gold exploitation and malaria occurrence. Specifically, clusters 2, 3 and 4 for *Plasmodium falciparum* and 1 and 4 for *Plasmodium vivax*, are located around medium and high gold exploitation areas in the country (Fig 13). Furthermore, all the central municipalities highlighted in Tables 3 and 2 (except for La Pedrera and Tarapacá in Amazonas) are located in the departments: Antioquia, Bolívar, Chocó, Córdoba, Valle del Cauca, Cauca and Nariño, which are precisely where gold-mining is most prevalent [85].

## 6 Discussion and conclusions

Malaria in Colombia was characterized by a different intensity, connectivity and segregation in each region. While there was a general pattern of risk throughout the country associated with occupational hazard, some populations experienced intense malaria exposure in endemic pockets. Understanding the interaction of such pockets is fundamental for designing appropriate malarial control strategies. Here we have produced a systematic approach that analyzes malaria under three dimensions: Clustering, Synchrony and Ethnicity.

Our findings suggest that controlling the epidemic in the lower Cauca Basin and the Caribbean had significant effects throughout the country. The reduction of *Plasmodium vivax* was followed by an increase of the same parasite, in connected clusters, in the Amazon Basin and the Central Pacific region. The increase of *Plasmodium vivax* was observed in locations that also experienced sustained and moderate deforestation throughout the territory. We find that epidemic and endemic characteristics coexist in infection by *Plasmodium vivax* and *Plasmodium falciparum*. While we did not study the effect of one population on the other, further studies can explore if the reduction of cases in the Caribbean and lower Cauca Basin were first experienced in the epidemic (“other”) or endemic (“indigenous”) populations. Similarly, it would be interesting to explore if the increase in *Plasmodium vivax* and *Plasmodium falciparum* of the Pacific region was first experienced by the indigenous, Afrocolombian, or population with no ethnic denomination. It is possible that the endemic indigenous population has an asymptomatic parasite carriage which, as previous studies suggest [42], might contribute strongly to malaria transmission and progression in the mentioned areas. Further studies, including genetic samples, will confirm if these populations in fact show asymptomatic behavior.

We also find that habitat transformation intensity harbors or reduces intensity of malarial infection. In line with reports from other locations in Latin America [43], we find that moderate deforestation fosters malarial infection of both parasites, and our findings suggest that urbanization was associated with some of the most important reductions of *Plasmodium vivax* infection in the Caribbean. Thus, malaria control strategies should pay particular attention to habitat transformation in the sense that slow and steady habitat transformation of large territories may be conducive to increases in the burden of malarial infection, while a moderate process of urbanization in hotspots, can have drastic effects not only within the region, but it may also have both positive and negative effects elsewhere. These strategies could also contemplate gold exploitation as an incidence factor, since both our findings and several studies show a correlation between gold-mining areas and malaria. [85] [44]

Our findings also suggest that the epidemic changes of malarial infection in Colombia are complex, comprised of a multiplicity of interconnected trends by parasite and region. This is consistent with the unstable transmission pattern exhibited in countries with rapid reductions of malaria. We observe a 5 year period between peaks of transmission for *Plasmodium vivax*, while epidemics of *Plasmodium falciparum* have been less intense, but more frequent (3 years). Control strategies should not only aim to adapt and mitigate to current burdens of malarial infection, but also be prepared for the re-introduction of malaria in locations where it was previously not endemic or epidemic. Such increases are to be expected in areas with sustained and moderate deforestation and without dense human occupation.

Our findings have potential implications for malarial infection control. First, we found that malaria in Colombia did present different, isolated pockets with distinctive epidemic characteristics. These hotspots play an important role in malaria transmission and when targeted, constitute an efficient way to reduce malaria intensity [86]. Furthermore, the magnitude of such differences in epidemic characteristics is relevant in studying the pressure of anti-malarials upon the parasite, since the emergence of resistance has been reported in the country. We found that *Plasmodium falciparum* was particularly acute among the Afrocolombian population of the Pacific region, while in the Cauca Basin, it constituted an isolated outbreak of the population with no ethnic denomination, while in the Pacific, the outbreak was dispersed among both the Afrocolombian and the indigenous populations.

Different parasite loads among ethnically and culturally distinct populations constitute the quintessential mechanism of selective pressures that are ideal for the evolution of parasites. The diversity of epidemic characteristics of malarial infection among the subpopulations of Colombia account for an ideal environment for parasite evolution, where plasmodia persist under different pressures of asymptomatic individuals, susceptible classes of ethnically distinct populations, and public health interventions using different anti-malarial strategies. Such diversity provides the necessary conditions, acting as isolated experiments, and then sharing “successful” results, for the emergence of resistant parasites.

Second, the patterns of endemicity observed in these populations suggested that prevention efforts should be population specific, and vary according to the epidemic characteristics exhibited by the parasite in the targeted population. Demographic, social and population factors confound studies limited to environmental factors and are also excluded from many studies [87] and thus could hold important insight on malaria control. We are currently experiencing an epidemic transition in malarial infection in Colombia, where parasite loads in populations are being transformed (potentially as populations are also subject of change). To understand such transitions, a systematic method to classify malarial infection in terms of vulnerability of populations over space, and the variation of those epidemic characteristics over time is crucial to implement public health strategies.

Therapeutic failures have been suggested to be correlated with high intestinal parasite loads [19]. The effectiveness of bed nets has been reported to be low among populations that experience intense malaria exposure [32]. We have identified populations that experienced malaria endemicity, where prevention efforts focused on the distribution of bed nets. Our findings, combined with previous knowledge suggest that public health interventions should integrate two aspects: 1) Diagnostic and treatment of asymptomatic malaria; and 2) Prevention strategies for occupational hazard.

Third, prevention strategies focusing on populations with endemic malaria would yield a reduction of occupational hazard malaria, since both types of epidemic characteristics exist in the same clusters of malaria, and interact in ways that determine their spatio-temporal epidemic characteristics.

### 6.1 Limitations

Our study was constrained by data availability at the spatial and temporal resolutions we observed for the epidemiological data. We are aware that it would have been ideal to include variables that describe vector ecology, distribution and behavior, pathogen genetic information, climatic variation during the study period, and other sociodemographic information. Further research will show whether including these variables alters our findings. It is possible that such information will improve the ability of the model to correctly identify clusters, or more interestingly, the overlapping of clusters and variables may shed light into ongoing debates, such as vector displacement, for example. Our findings should be cautiously interpreted, but we are confident that they inform policy in a way that few other epidemiological studies currently advise, and furthermore, our findings are complementary to vector, pathogen and epidemic literature, to improve our understanding of malarial infection dynamics in Colombia at a national level, and the way in which such dynamics interact at a regional level.

## 7 Additional figures

**Fig 14:**
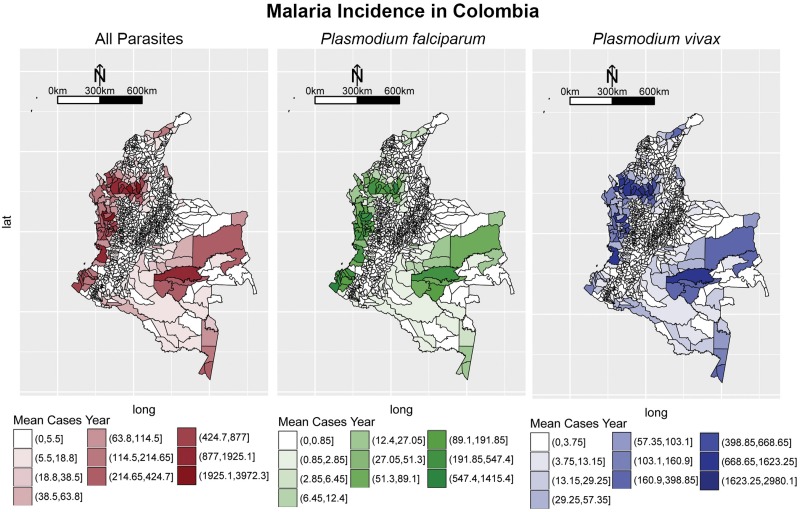
Malarial incidence for both species in Colombia from 2007-2015. Intervals where constructed using the Jenks procedure [88].

For completeness, we include the malaria incidence extracted from the data in Fig 14.
